# *Staphylococcus aureus* α-hemolysin induces DNA methylation changes in human Th1 cells

**DOI:** 10.1007/s12026-025-09647-0

**Published:** 2025-06-11

**Authors:** Iwona Karwaciak, Joanna Pastwińska, Anna Sałkowska, Kaja Karaś, Marta Sobalska-Kwapis, Jarosław Dastych, Marcin Ratajewski

**Affiliations:** 1 Laboratory of Epigenetics, Institute of Medical Biology, Polish Academy of Sciences, Lodowa 106, Lodz, 93-232 Poland; 2 Centre for Digital Biology and Biomedical Science - Biobank Lodz®, Faculty of Biology and Environmental Protection, University of Lodz, Lodz, 91-402 Poland; 3 Laboratory of Cellular Immunology, Institute of Medical Biology, Polish Academy of Sciences, Lodowa 106, Lodz, 93-232 Poland

**Keywords:** α-Hemolysin, Th1, *Staphylococcus aureus*, DNA methylation

## Abstract

α-Hemolysin is one of the most dangerous virulence factors produced by *Staphylococcus aureus*. Among the immune cells that respond to this pathogenic bacterium, Th1 lymphocytes play a critical role. In our study, we investigated the impact of α-hemolysin on the methylation of the Th1 cell genome. Our findings revealed that α-hemolysin upregulates HELLS and DNMT3A expression while downregulating DNMT3L expression at the protein level. Whole-genome bisulfite sequencing analysis revealed significant alterations in DNA methylation, particularly in regions outside CpG sequences. These results suggest that bacterial proteins can act as potent epigenetic modulators in human cells, influencing their activity, plasticity, and phenotype.

**Supplementary information:**

The online version contains supplementary material available at 10.1007/s12026-025-09647-0.

## Introduction

*Staphylococcus aureus* is a common bacterium that naturally resides on human skin and in nasal passages; although typically harmless in these locations, it can become highly pathogenic when introduced into wounds or the bloodstream. *S. aureus* is a major contributor to skin and soft tissue infections, surgical-site infections, and sepsis, and it poses a significant public health challenge, causing widespread disease in community environments and functioning as a prominent healthcare-associated pathogen in medical facilities [1–3]. One of the key virulence factors of this bacterium is α-hemolysin, also known as α-toxin, a member of the pore-forming beta-barrel toxin family. This toxin is released in a monomeric form and, upon binding to the cellular membranes of target cells, assembles into a heptameric pore. This pore induces cell death through apoptosis and necrosis at relatively high concentrations [4–6]. However, at sublytic concentrations, α-hemolysin impacts immune responses, alters cellular signaling, and, as recent studies have demonstrated, modulates the epigenetic programming of cells [7–10].

CD4+ T lymphocytes appear to play a critical role in protecting the body against *S. aureus* infections, with Th1 and Th17 subsets being particularly important [2, 3, 11, 12]. In our previous work, we investigated the impact of α-hemolysin on Th17 lymphocytes and demonstrated that the toxin influences the expression of numerous genes associated with epigenetic regulation in these cells [10]; this, in turn, led to alterations in the methylation patterns within their genomes. Inspired by these findings, we conducted a similar analysis on differentiating Th1 lymphocytes. Analysis of methyltransferases at the protein level revealed that this toxin increases the protein levels of HELLS, DNMT3A and decreases those of DNMT3L. We propose that alterations in these proteins are associated with changes in methylation at the CG, CHG and CHH sites, as demonstrated by whole-genome bisulfite sequencing (WGBS).

## Materials and methods

### Reagents

Active recombinant *S. aureus* α-hemolysin (ab233724) was purchased from Abcam (Cambridge, UK), and active recombinant *S. aureus* α-hemolysin (Hla-729S) was purchased from Creative Biomart (Shirley, NY, USA). Hemolysin from Abcam was used in the cell viability assays, three RNA-seq experiments and three WGBS-seq experiments, whereas the product from Creative Biomart was used in the cell viability assays, two RNA-seq experiments and two WGBS-seq experiments, as well as in the qPCR, western blotting, and ChIP assays.

### Cell viability

The effect of α-hemolysin on the viability of CD4+ cells differentiating into Th1 cells was evaluated via the CellTiter-Glo® Luminescent Cell Viability Assay (Promega Corporation, Madison, WI, USA) following the manufacturer’s protocol. Luminescence measurements for each well in the 96-well plate were obtained with an Infinite® 200 PRO microplate reader (Tecan, Männedorf, Switzerland).

### Isolation of CD4+ cells and Th1 differentiation

Naïve CD4+ T cells were isolated from the buffy coats of healthy, anonymous donors obtained from the Regional Center for Blood Donation and Blood Treatment in Lodz, Poland, via CD4 M-pluriBead® anti-human beads (pluriSelect Life Science, Leipzig, Germany). These cells were then differentiated into Th1 cells over a 5-day period via a Human Th1 Cell Differentiation Kit (R&D Systems, CDK001, Minneapolis, MN, USA) following the manufacturer’s protocol. The differentiation medium comprised RPMI 1640 supplemented with 5% fetal bovine serum (FBS), 2 mM L-glutamine, 50 U/mL penicillin, 50 μg/mL streptomycin, and 50 μM 2-mercaptoethanol (2-ME), along with Human Th1 Reagent 1 and Human Th1 Reagent 2 from the differentiation kit as described previously [13].

### RNA-seq and data analysis

Total RNA was extracted from primary CD4+ cells that had been induced to differentiate into Th1 cells over a 5-day period in the presence of 75 ng/mL α-hemolysin. The RNA libraries were prepared as previously described in our earlier study [10]. These libraries were subjected to high-resolution RNA sequencing (RNA-seq) via the NovaSeq 6000 platform (Illumina) at Novogene (Cambridge, UK).

High-quality reads were obtained by filtering out sequences containing adapter contamination, poly-N stretches, or low-quality bases. The retained paired-end reads were aligned to the GRCh38 reference genome via HISAT2 v2.0.5 [14]. Following alignment, transcript assembly was conducted with StringTie v1.3.3b [15]. Gene expression levels were quantified via FeatureCounts v1.5.0-p3 [16]. To identify differentially expressed genes (DEGs), we employed the DESeq2 R package v1.20.0 [17]. Multiple testing correction was performed via the Benjamini–Hochberg method to control the false discovery rate (FDR). Genes exhibiting an absolute log2-fold change (|log2 FC|) of at least 1 and an adjusted p value of ≤ 0.05 were classified as differentially expressed.

The RNA-seq dataset is publicly accessible under BioProject accession number PRJNA1254664 in the NCBI Short-Read Archive (SRA) https://www.ncbi.nlm.nih.gov/bioproject/PRJNA1254664. ShinyGO version 0.77 was used to conduct functional annotation of DEGs through Gene Ontology (GO) analysis and Kyoto Encyclopedia of Genes and Genomes (KEGG) pathway enrichment [18, 19].

### Real-time RT–PCR

Total RNA was isolated via TRI Reagent (Molecular Research Center, Cincinnati, OH) and suspended in nuclease-free water. A 5 μg aliquot of the RNA was used for cDNA synthesis, following the manufacturer’s protocol with the Maxima First-Strand cDNA Synthesis Kit (Thermo Scientific/Fermentas, Vilnius, Lithuania). Quantitative real-time PCR (RT–qPCR) was performed on a LightCycler 480 system (Roche, Basel, Switzerland) with SYBR Green I Master Mix (Roche). The PCR conditions were as described previously [10]. mRNA levels were normalized via a geometric mean approach based on three housekeeping genes: hypoxanthine phosphoribosyltransferase 1 (*HPRT1*), hydroxymethylbilane synthase (*HMBS*), and ribosomal protein L13A (*RPL13A*) according to the procedure described by Vandesompele et al. [20]. The primer pairs used in this study can be found in Table S1.

### Western blotting

Human primary CD4+ cells were cultured in 6-well plates at a density of 12 × 10^6^ cells per well and differentiated into Th1 lymphocytes with 75 ng/mL α-hemolysin for 5 days. The cells were then harvested and lysed as previously described [21]. The antibodies used included anti-DNMT1 (D63A6) (#5032), anti-DNMT3A (D23G1) (#3598), anti-DNMT3L (E1Y7Q) (#13,451), anti-HELLS (#7998), anti-tri-methyl-histone H3 (Lys36) (D5A7) (#4909), and anti-β-actin [13E5] (#4970), which were obtained from Cell Signaling Technology (Danvers, MA, USA). Specific protein bands were visualized via SuperSignal West Pico Chemiluminescent Substrate (Thermo Fisher Scientific, Waltham, MA, USA) and imaged via a G:Box chemiluminescence system (Syngene, Cambridge, UK). Densitometry of the resulting bands was performed via ImageJ (http://imagej.nih.gov/ij/).

### Whole-genome bisulfite sequencing (WGBS)

WGBS was conducted on DNA extracted from CD4+ cells, which were isolated and differentiated into Th1 lymphocytes in the presence of 75 ng/mL α-hemolysin, sourced from five distinct human donors. The sequencing was performed at Novogene’s Cambridge Sequencing Centre, which is located in Cambridge Science Park, UK. The complete sample processing and analysis methodology is comprehensively detailed in our prior publication [10].

Differentially methylated regions (DMRs) were identified via the DSS software package [https://www.bioconductor.org/packages/release/bioc/html/DSS.html], which employs a dispersion shrinkage method for detecting differential methylation in bisulfite sequencing data [22–24]. Genes associated with these DMRs were determined based on their genomic locations, specifically considering overlaps with either the gene body region—defined as the span from the transcription start site (TSS) to the transcription end site (TES)—or the promoter region, defined as the 2 kilobases upstream of the TSS. The WGBS data have been deposited in the NCBI Short Read Archive (SRA) under BioProject accession number PRJNA1146149, https://www.ncbi.nlm.nih.gov/bioproject/PRJNA1146149. Functional enrichment analyses of the DMR genes were conducted via GOseq, which uses Wallenius noncentral hypergeometric distribution as the sampling method [25]. Statistical significance was defined as p ≤ 0.05.

### Chromatin immunoprecipitation (ChIP)

To assess whether α-hemolysin induces H3K36me3 occupancy at the differentially methylated regions of the *LAMC1* (chr1:183,022,904–183,023,040), *ZC3H12C* (chr11:110,092,805–110,092,908), and *LTBP3* (chr11:65,558,311–65,558,465) genes in Th1 cells, chromatin immunoprecipitation (ChIP) was performed via the EZ-Magna ChIP A/G Kit (EMD Millipore, Billerica, MA, USA) following the manufacturer’s protocol. Normal mouse IgG (EMD Millipore) was used as a control, while an anti-tri-methyl-histone H3 (Lys36) (D5A7) (#4909; Cell Signaling Technology) antibody was used to specifically target H3K36me3. The relative enrichment of *LAMC1*, *ZC3H12C*, and *LTBP3* was quantified via real-time PCR. The amplification conditions were as follows: initial denaturation at 95 °C for 10 min, followed by 40 cycles of 95°C for 20 s, 58°C for 20 s, and 72°C for 20 s. The primers used were as follows: *LAMC1* (forward: 5′-GCTTCACGAAACTCATCTGGC-3′, reverse: 5′-CTCCCGAACAGAGGTCCCG-3′), *2C3H12C* (forward: 5′-TTTCAGTTCTCGGCGTTCTCC-3′, reverse: 5′-CCCTCTTTTCCTTCTCCTCCG-3′), and *LTBP3* (forward: 5′-AAGTTGAGGCGGAGAGGAGG-3′, reverse: 5′-CAGCCAGGGAAAACAACTGC-3′). The abundance of specific sequences was calculated via the ΔCt method, with the Ct value of the input DNA used as the reference. The relative level of sequences in the samples was determined via the formula 1000 * 2^(-ΔCt), where ΔCt = Ct(sample)—Ct(input DNA), as described previously [26].

### Statistical analysis

The data were analyzed via Friedman one-way repeated-measures ANOVA by ranks, followed by a Student–Newman–Keuls post hoc test. The analyses were conducted via SigmaStat v4.0 (Systat Software, San Jose, CA). Statistical significance was defined as p ≤ 0.05.

## Results and discussion

**Fig. 1:**
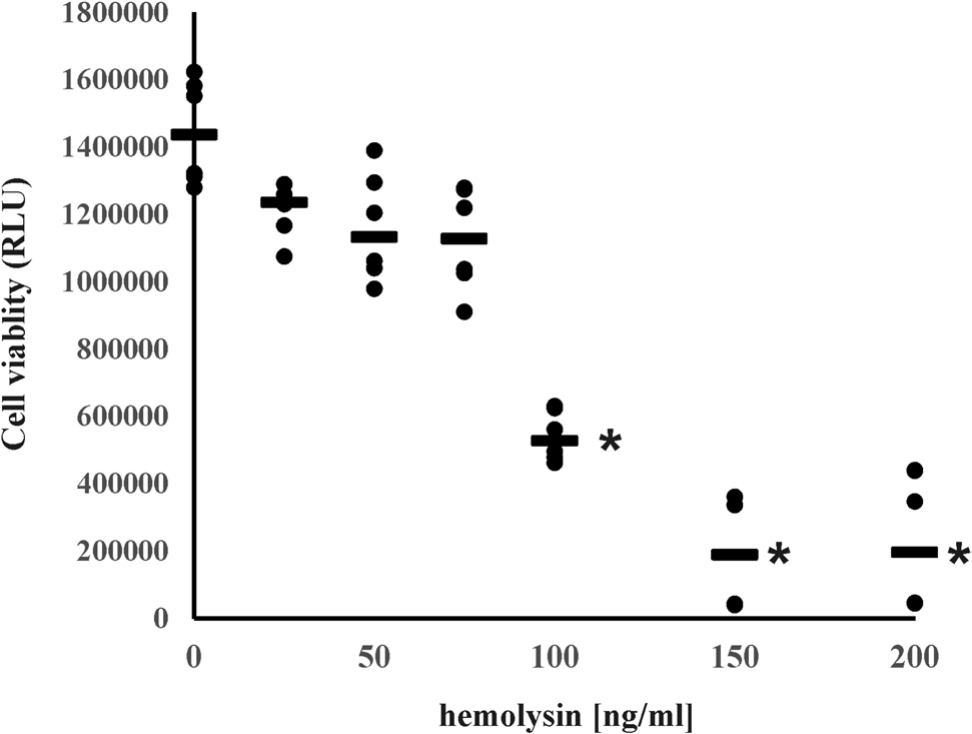
The impact of α-hemolysin on the viability of CD4+ T cells differentiating into human Th1 lymphocytes was investigated. CD4+ cells from six different donors were exposed to increasing concentrations of α-hemolysin and differentiated into Th1 lymphocytes over a period of five days. Cell viability was then assessed via the CellTiter-Glo® Luminescent Cell Viability Assay (Promega). The results are presented as statistical dot plots, with median values indicated by bars (n = 6); * indicates statistical significance at *p* < 0.05

In the initial stage of our research, we investigated how α-hemolysin affects the viability of CD4+ T cells differentiating into Th1 lymphocytes. We found that concentrations above 75 ng/mL were significantly toxic to these cells (Fig. 1), corroborating previous studies that demonstrated that this T lymphocyte subpopulation is far more sensitive to α-hemolysin than Th17 cells are [10, 27].

**Fig. 2:**
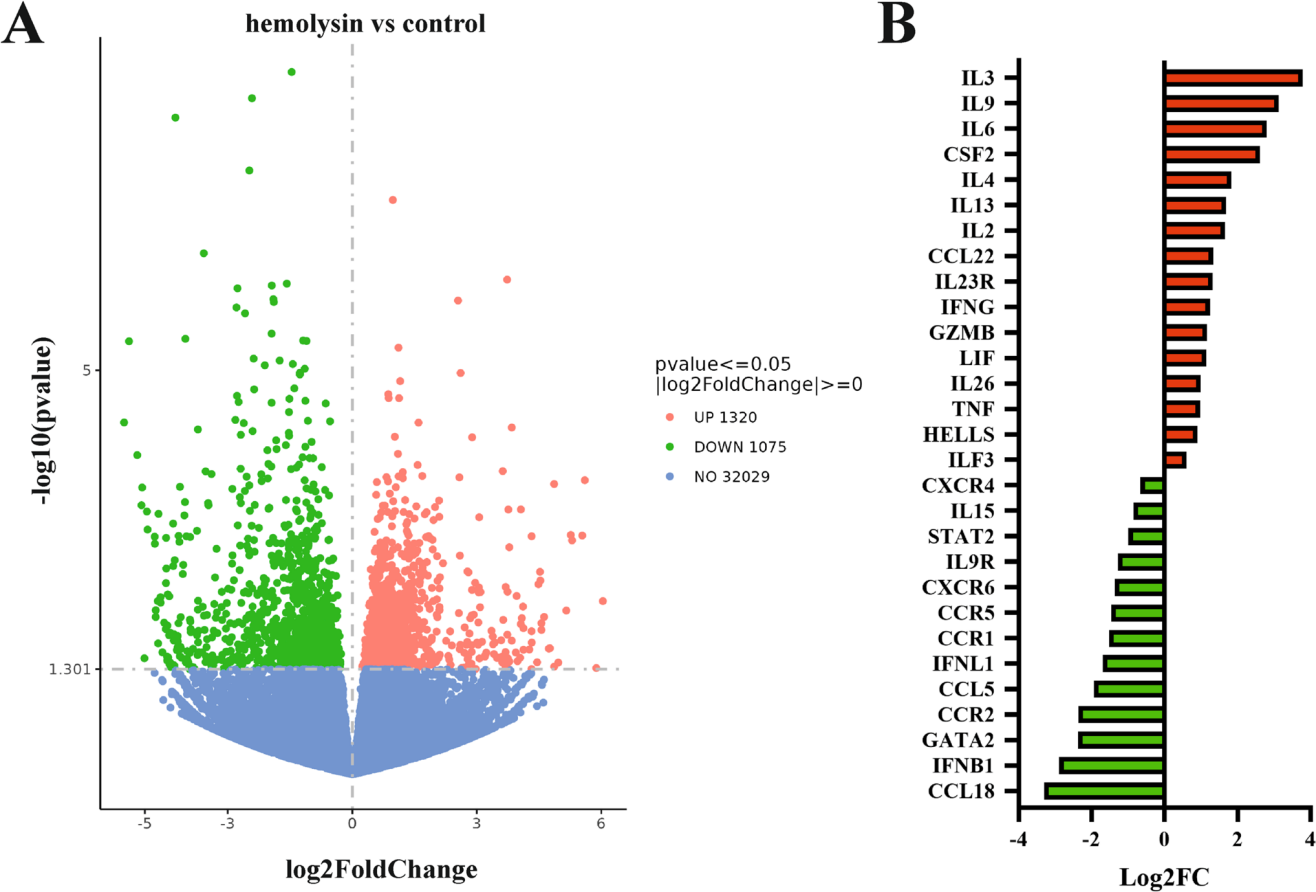
RNA-seq analysis demonstrated that α-hemolysin modulates the transcriptomic profile of human Th1 cells. (**A**) Volcano plot showing the differentially expressed genes (DEGs) in CD4+ cells that differentiated into Th1 cells upon treatment with 75 ng/mL α-hemolysin. The red dots represent upregulated genes, the green dots indicate downregulated genes, and the blue dots denote genes whose expression did not significantly change. (**B**) Compilation of genes involved in immune system regulation, showing altered expression in differentiating Th1 cells following α-hemolysin treatment, as determined by RNA-seq analysis of data from five independent donors (n = 5)

**Fig. 3:**
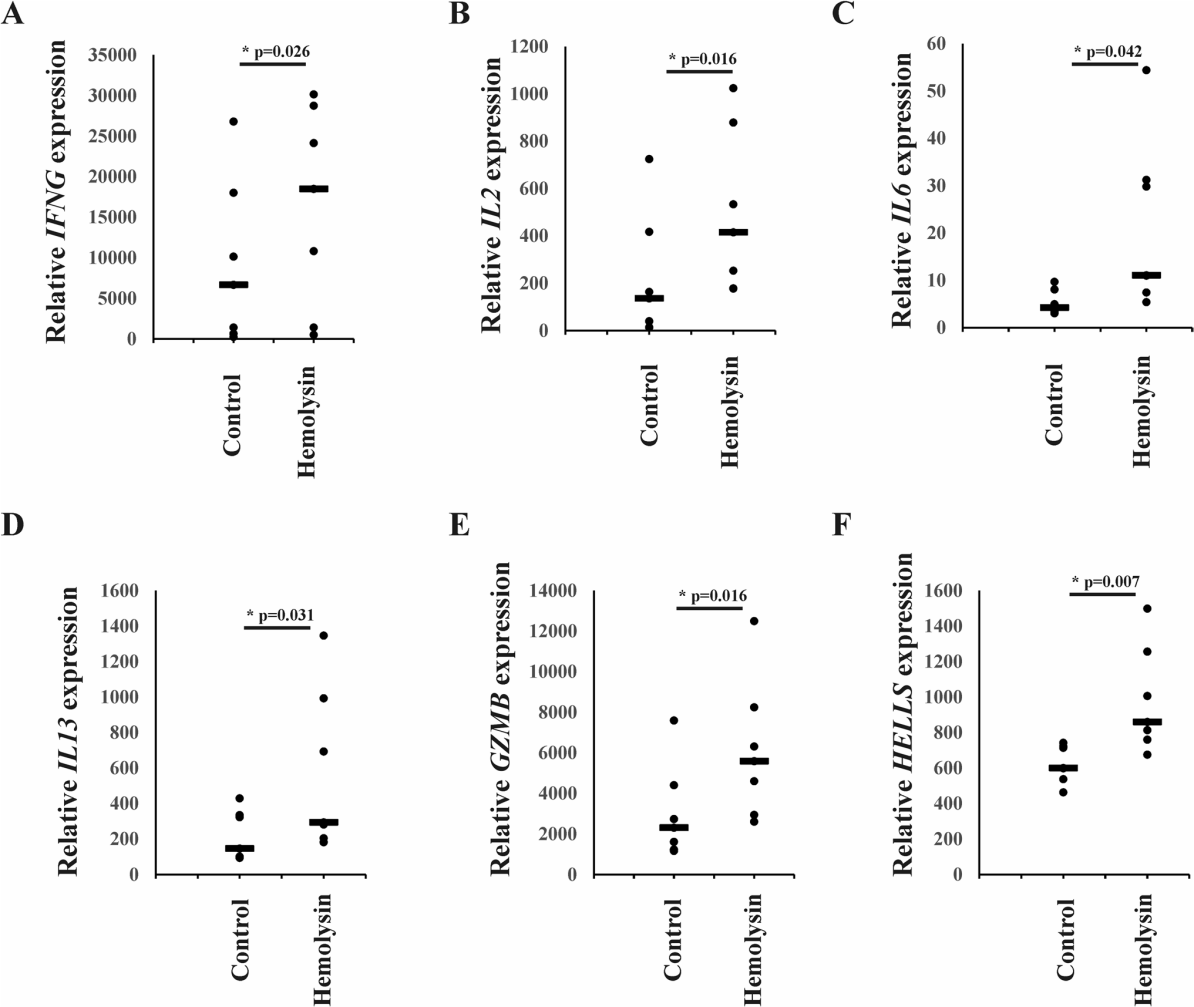
Effect of α-hemolysin on the expression of selected genes in differentiating Th1 lymphocytes. CD4+ cells isolated from the buffy coats of anonymous donors were differentiated for 5 days in the presence of α-hemolysin (75 ng/mL). Real-time quantitative PCR was performed to measure the expression levels of *IFNG*, *IL2*, *IL6*, *IL13*, *GZMB*, and *HELLS*. The results, presented as a dot plot with median values, were derived from seven independent donors (n = 7), with statistical significance indicated

**Fig. 4:**
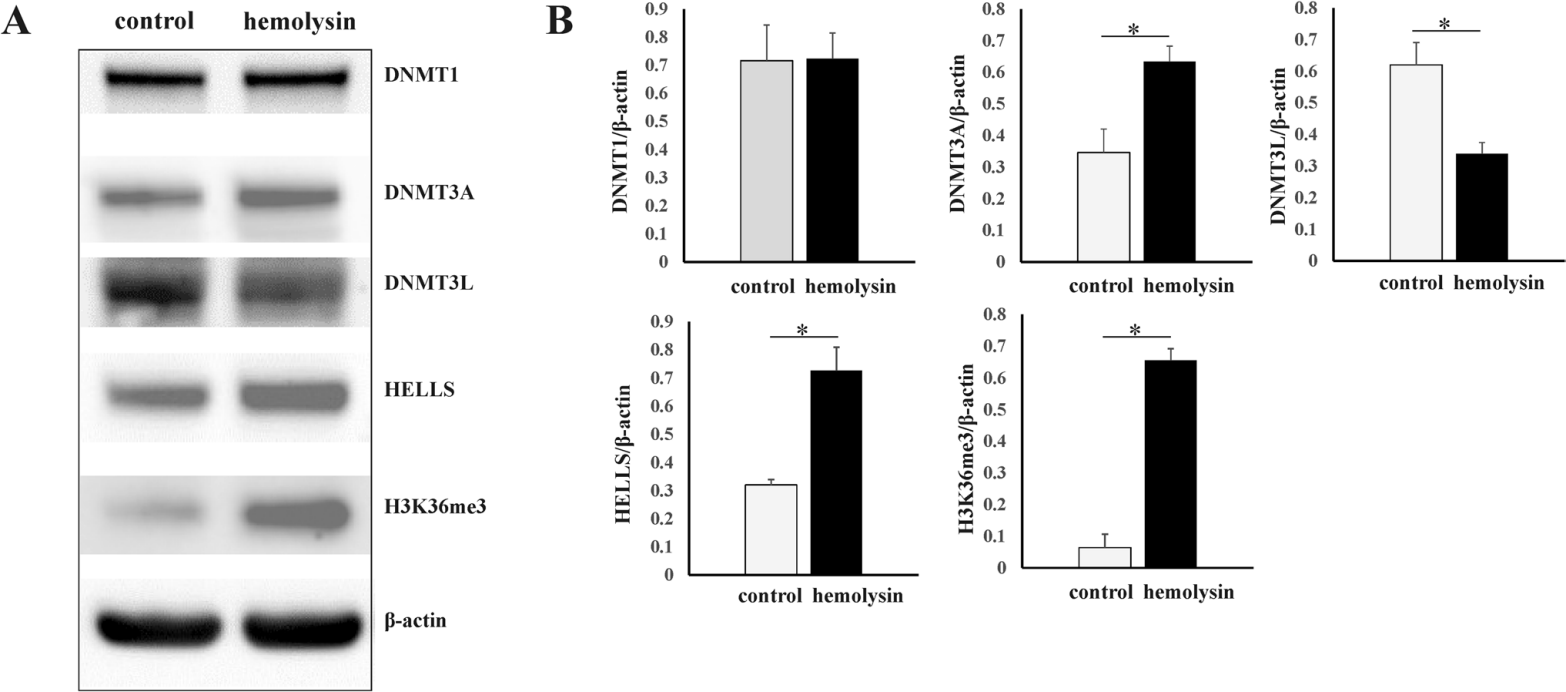
The impact of α-hemolysin on the expression of key proteins involved in DNA methylation during Th1 lymphocyte differentiation was investigated. CD4+ cells isolated from the buffy coats of anonymous donors were cultured in the presence of 75 ng/mL α-hemolysin for five days. Afterward, the cells were harvested, lysed, and analyzed via western blotting. (**A**) The protein expression levels of DNMT1, DNMT3A, DNMT3L, HELLS, H3K36me3, and β-actin were assessed. (**B**) Densitometric analysis of DNMT1, DNMT3A, DNMT3L, HELLS, and H3K36me3 expression in 3 different donors (n = 3). The results are shown as the means ± S.D.s. * denotes statistical significance at *p* < 0.05

**Fig. 5:**
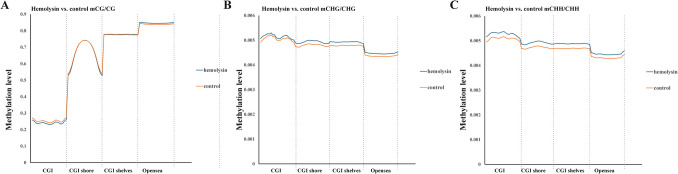
α-Hemolysin modifies the methylation levels across various functional genomic regions of human Th1 lymphocytes, as revealed by WGBS. Graphs comparing the α-hemolysin-treated samples with the controls, illustrating the mCG/CG (**A**), mCHG/CHG (**B**), and mCHH/CHH (**C**) ratios. CD4+ cells were isolated from the buffy coats of anonymous donors and cultured with α-hemolysin (75 ng/mL) for 5 days to promote Th1 differentiation. Afterward, the cells were harvested, lysed, and processed for DNA isolation, bisulfite treatment, and WGBS. The data were obtained from five individual donors

**Fig. 6:**
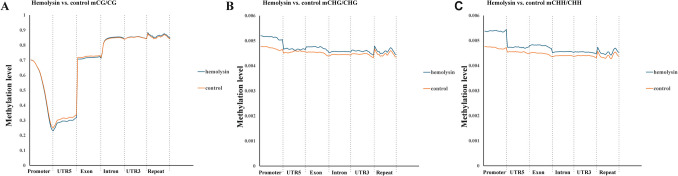
α-Hemolysin impacts the methylation levels of various gene regions in human Th1 lymphocytes, as demonstrated by WGBS. The graph displays the mCG/CG (**A**), mCHG/CHG (**B**), and mCHH/CHH ratios (**C**) across gene regions. CD4+ cells were isolated from the buffy coats of anonymous donors and cultured with α-hemolysin (75 ng/mL) for 5 days to promote Th1 differentiation. The cells were then harvested, lysed, and processed for DNA isolation, bisulfite treatment, and WGBS. The data were collected from five independent donors

**Fig. 7:**
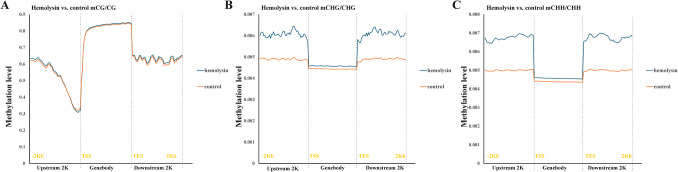
α-Hemolysin influences the methylation levels within gene bodies and their adjacent regions in human Th1 lymphocytes, as shown by WGBS. The graph presents the mCG/CG (**A**), mCHG/CHG (**B**), and mCHH/CHH ratios (**C**) across gene bodies and flanking regions (2 kb upstream and 2 kb downstream). CD4+ cells were isolated from the buffy coats of anonymous donors and cultured with α-hemolysin (75 ng/mL) for 5 days to induce Th1 differentiation. Next, the cells were harvested, lysed, and processed for DNA isolation, bisulfite treatment, and WGBS. The data were collected from five independent donors

**Fig. 8:**
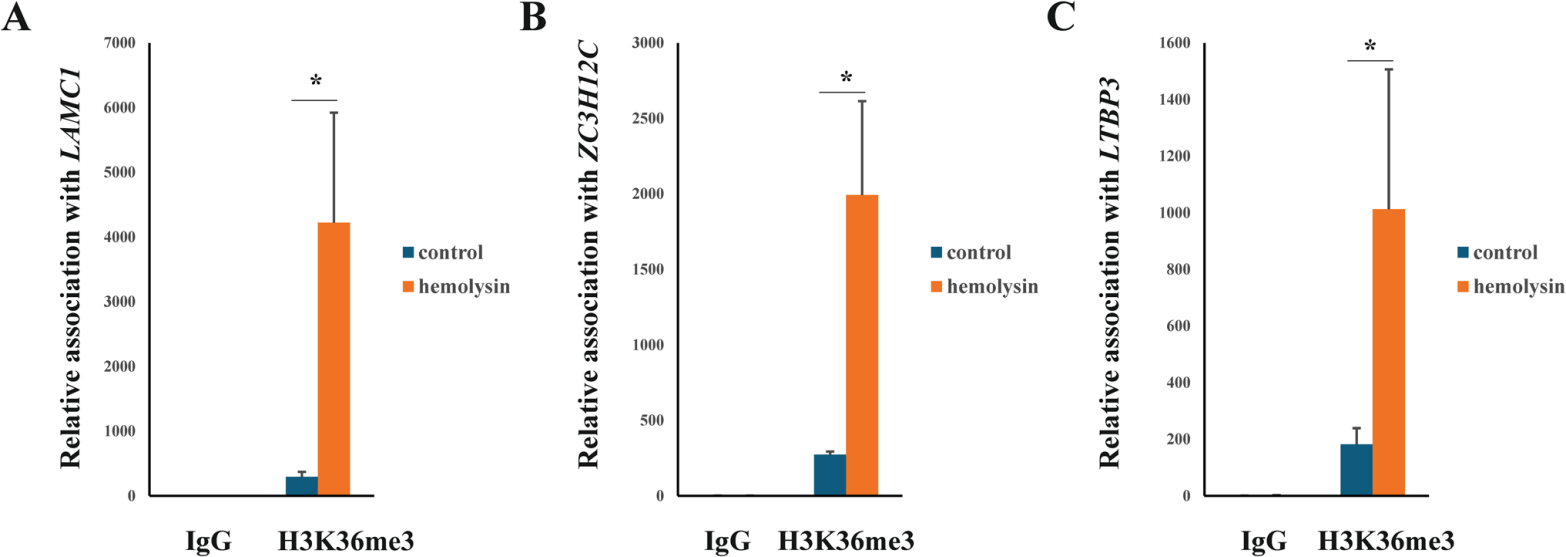
α-Hemolysin (75 ng/mL) promotes the enrichment of H3K36me3 at the identified DMR regions of the *LAMC1*, *ZC3H12C*, and *LTBP3* genes, as demonstrated by chromatin immunoprecipitation in CD4+ lymphocytes that differentiated into Th1 cells over 5 days. The results are presented as the mean ± S.D. (n = 5). * indicates a statistically significant difference at *p* < 0.05

**Fig. 9:**
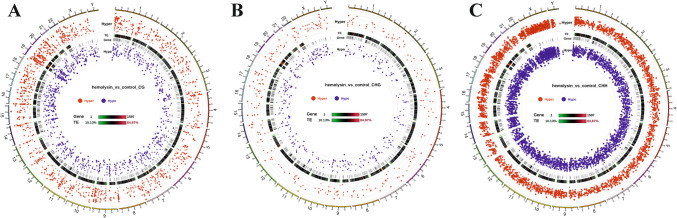
Circos plots showing the significance and distribution of DMRs on specific chromosomes in the CG (**A**), CHG (**B**) and CHH (**C**) contexts. Hyper and hypo-DMR statistical values: log5(|areaStat|). The higher and larger the point is, the larger the difference between the two groups. TE indicates the heatmap of the percentage of repeat elements. The genes indicate the heatmap of gene density

**Fig. 10:**
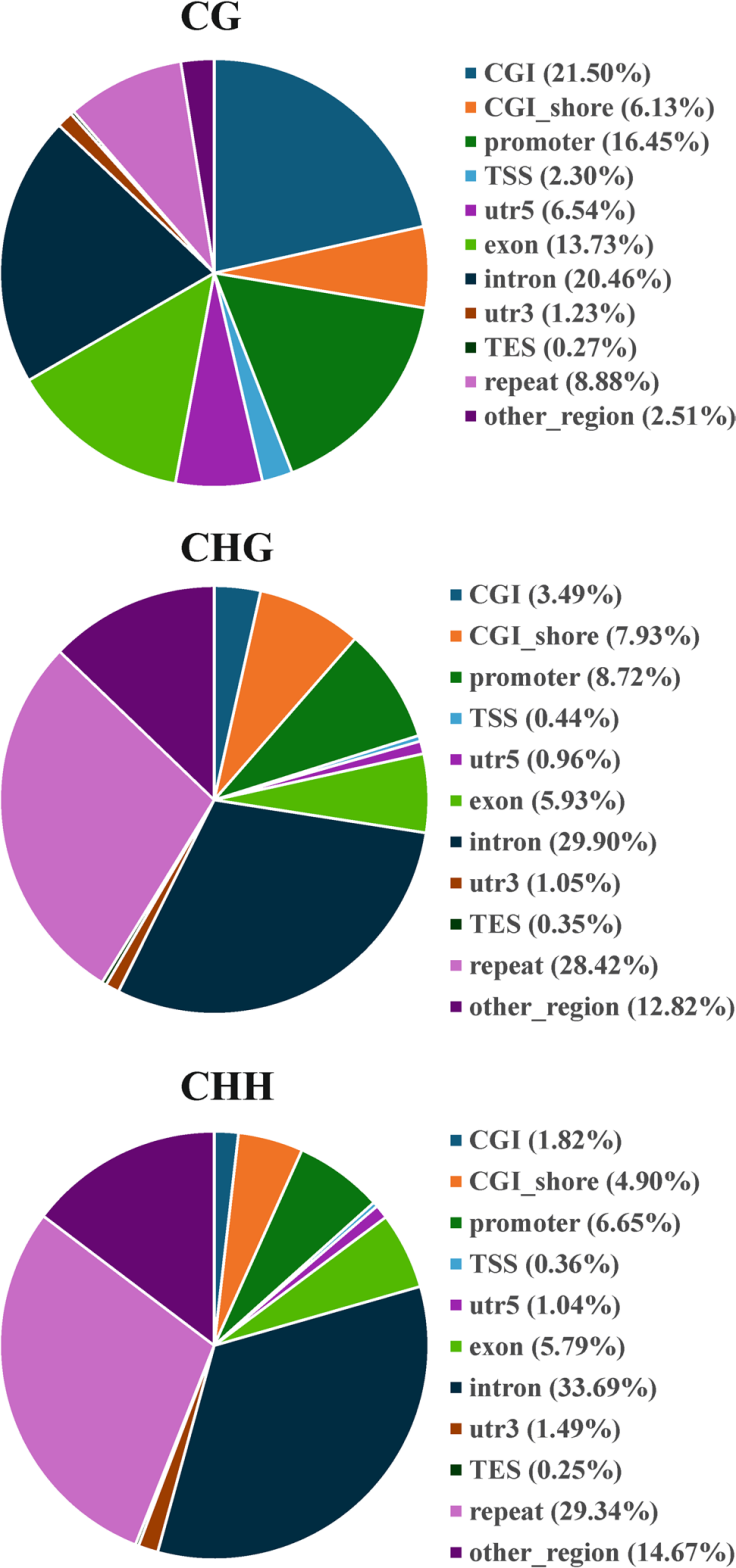
Ratio of DMRs in gene functional regions modified by CGs, CHGs, and CHHs in Th1 lymphocytes

Next, we performed transcriptomic analysis of Th1 cells via samples from several healthy human donors. This analysis revealed that α-hemolysin alters the expression of more than 2,300 genes (Fig. 2A), many of which are associated with immune responses. These include interleukins such as *IFNG*, *IL2*, *IL4*, *IL6*, *IL13*, *IL15*, and *IL26*; transcription factors such as *GATA2* and *STAT2*; and receptors such as *CCR1*, *CCR2*, and *CCR5* (Fig. 2B and DataSet1). Gene Ontology (GO) analysis revealed numerous terms associated with the regulation of translation, RNA processing, mitochondrial and organelle inner membrane functions, and nucleotide binding (Table S2). KEGG pathway analysis of differentially expressed genes (DEGs) revealed disruptions in pathways such as 'DNA replication', 'ribosome biogenesis', 'nucleotide metabolism', and 'cytokine–cytokine receptor interaction' in cells treated with α-hemolysin (Table S3). Interestingly, consistent with our previous study on Th17 lymphocytes [10], we identified *HELLS* as one of the differentially expressed genes (DEGs) (Fig. 2B), which was upregulated following α-hemolysin treatment. However, we did not detect any other genes directly involved in DNA methylation. To validate these findings, we conducted quantitative PCR to examine whether α-hemolysin indeed influences the expression of selected genes. We confirmed the upregulation of *IFNG*, *IL2*, *IL6*, *IL13*, *GZMB* and *HELLS* (Fig. 3). The induction of *IFNG* following α-hemolysin treatment in Th1 cells is consistent with the findings of a previous study by Breuer et al., suggesting that α-hemolysin might enhance the proinflammatory properties of Th1 lymphocytes [28]. Our earlier observations in Th17 cells, where α-hemolysin decreased protein levels despite transcriptional upregulation, as observed with DNMT3B [10], prompted us to perform western blot analysis to evaluate the expression of proteins involved in the regulation of DNA methylation, including DNMT1, DNMT3A, DNMT3B, DNMT3L and HELLS. Indeed, western blot analysis of these proteins confirmed the upregulation of HELLS and DNMT3A in Th1 lymphocytes exposed to α-hemolysin (Fig. 4) and the decrease in DNMT3L levels. Interestingly, in contrast to Th17 lymphocytes, we were unable to detect the DNMT3B protein (data not shown). Since histone H3 trimethylated at lysine 36 (H3K36me3) is involved in the positioning of DNMT3s [29–31], we also analyzed this mark in cells treated with α-hemolysin. As expected, a significant increase in this modification was observed in cells exposed to the toxin (Fig. 4). This led us to investigate whether Th1 cells cultured with α-hemolysin also exhibit altered genomic methylation. To achieve this goal, we performed WGBS on DNA from five human donors. As shown in Table 1, we obtained between 81.57 and 111.88 GB of clean bases, with a bisulfite conversion rate above 99.60% for each sample. The mapping rate to the reference genome ranged from 85.69% to 90.21% (Table 1). We analyzed three types of methylation contexts: CG, CHG, and CHH. On average, 98.85% mCG, 0.26% mCHG, and 0.87% mCHH were detected in the control lymphocytes, whereas 98.81% mCG, 0.28% mCHG, and 0.90% mCHH were detected in the α-hemolysin-treated cells (Fig. S1). We then examined the distribution of methylation across different genomic regions, including CG islands (CGIs), CGI shores, CGI shelves, and open sea regions. As shown in Fig. 5A, we observed a slight decrease in CG methylation levels within CGI regions in α-hemolysin-treated cells, along with a modest increase in methylation in the open sea regions (ranging from 1 to 5%). However, α-hemolysin treatment resulted in increased CHG and CHH methylation levels across all analyzed regions (ranging from 2 to 4%) (Fig. 5B, C). Next, we examined methylation changes in different gene functional regions. In the CG context, there was a decrease in methylation levels in 5’-UTRs and exons, whereas a slight increase was noted in repeated sequences (Fig. 6A) in cells cultured with α-hemolysin (ranging from 0.8 to 6.1%). In contrast, α-hemolysin led to increased CHG and CHH methylation levels in promoters, 5’-UTRs, exons, introns, 3’-UTRs, and repeated regions (ranging from 2 to 9%) (Fig. 6B, C). A detailed analysis of methylation levels in gene bodies and regions upstream and downstream of genes revealed a subtle increase in CG methylation levels in cells cultured with α-hemolysin (ranging from 0.6 to 1.4%) (Fig. 7A). The effect of α-hemolysin was more pronounced for CHG and CHH methylation in these regions, leading to significant hypermethylation both upstream and downstream of gene bodies (ranging from 3.2 to 33.3%) (Fig. 7B, C). Because we observed a significant increase in histone H3K36me3 modification following α-hemolysin treatment and since this modification serves as a platform for DNMT3s recruitment [29], we investigated the relationships between H3K36me3 enrichment and three differentially methylated regions (DMRs) located within the *LAMC1*, *ZC3H12C*, and *LTBP3* genes. Using chromatin immunoprecipitation (ChIP), we detected a marked increase in the association of H3K36me3 with these DMRs upon α-hemolysin treatment (Fig. 8). Interestingly, the *LAMC1* gene was among the differentially expressed genes (DataSet1) whose expression was upregulated following α-hemolysin treatment, despite increased methylation within its promoter region (Table S4); this could be explained by the fact that the CpG island associated with this gene is located outside the transcription unit and does not directly impact transcription [32] or that methylation of this CpG island reduces the binding of a transcriptional repressor, thereby promoting gene expression [33]. A total of 153 differentially expressed genes (DEGs) were found to have either hypermethylated or hypomethylated promoters (Table S4), and overall, 32 differentially expressed genes (DEGs) with hypermethylated promoters presented decreased expression, whereas 40 DEGs with hypomethylated promoters presented increased expression following α-hemolysin treatment. These findings suggest that promoter methylation may, at least in part, contribute to the regulation of the expression of these genes in response to *staphylococcal* α-hemolysin. We next analyzed the distribution of differentially methylated regions (DMRs) across specific chromosomes (Fig. 9 and DataSet2) and gene regions (Fig. 10). A total of 6,182 CG-type, 1,147 CHG-type, and 12,391 CHH-type DMRs in the gene region were identified. Similar to earlier findings, there were clear differences in the distribution of CG-type methylation compared with CHG and CHH methylation. The majority of CG-type DMRs were located in CGIs (21.5%), promoters (16.45%), exons (13.73%), and introns (20.46%). In contrast, most CHG-type DMRs were found in introns (29.90%) or repeated regions (28.42%). A similar pattern was observed for CHH-type methylation, with DMRs located primarily in introns (33.69%) and repeated regions (29.34%) (Fig. 10), which is in accordance with previous publications showing that non-CpG methylation occurs more frequently within gene bodies than in promoters [34]. We then conducted a gene ontology analysis of the DMR genes. This analysis revealed significantly enriched terms, including 'DNA binding'within the molecular function category, for the DMR genes in the CG context. Additionally, the significantly enriched terms in the biological process category included 'homophilic cell adhesion via plasma membrane adhesion molecules', 'cell–cell adhesion via plasma–membrane adhesion molecules', and 'cell–cell adhesion'. In the CC category, 'anchored component of the plasma membrane' was identified as an enriched term, whereas 'calcium ion binding' emerged as a significantly enriched MF term for the DMR genes in the CHH context (Table 2). Interestingly, no significantly enriched terms were identified for the DMR genes in the CHG context.

**Table 1:** WGBS sequencing data from the Th1 lymphocytes of five different donors

Donor	Samples	Raw Reads	Clean Reads	Clean Bases (G)	GC Content (%)	BS conversion rate	Mapped reads	Mapping rate (%)
7	Control	305,143,831	299,145,308	82.42	22.27	99.65	266,109,780	88.96
7	α-Hemolysin	341,648,720	334,023,536	91.61	22.4	99.632	290,834,389	87.07
8	Control	310,298,734	303,971,422	83.94	21.64	99.649	272,421,293	89.62
8	α-Hemolysin	358,657,088	349,423,075	95.79	21.59	99.644	299,407,503	85.69
11	Control	356,802,738	347,726,738	95.69	21.41	99.613	302,969,887	87.13
11	α-Hemolysin	304,598,170	298,868,691	82.53	21.09	99.683	269,295,070	90.1
12	Control	412,742,715	405,084,129	111.88	21.06	99.67	365,440,525	90.21
12	α-Hemolysin	353,704,992	347,152,138	95.85	21.73	99.673	312,871,009	90.13
13	Control	301,057,888	295,439,536	81.57	21.32	99.663	265,770,949	89.96
13	α-Hemolysin	321,020,351	314,666,781	86.9	20.82	99.673	282,877,326	89.9

**Table 2:** GOseq analysis results for the significant DMR genes in the CG and CHH contexts after α-hemolysin treatment in Th1 cells

Context	GO accession	Description	Term type	Overrepresented p value	Corrected p value	DMR item
CG	GO:0003677	DNA binding	Molecular function	0.0000092306	0.030138	70
CHH	GO:0007156	homophilic cell adhesion via plasma membrane adhesion molecules	Biological process	0.000011867	0.018131	38
CHH	GO:0098742	cell–cell adhesion via plasma-membrane adhesion molecules	Biological process	0.000011867	0.018131	38
CHH	GO:0098609	cell–cell adhesion	Biological process	0.000016659	0.018131	38
CHH	GO:0046658	anchored component of plasma membrane	Cellular component	0.000038503	0.031428	5
CHH	GO:0005509	calcium ion binding	Molecular function	0.000054192	0.035387	75

An increasing body of evidence suggests that bacteria and their proteins modulate gene expression in eukaryotic cells, including human cells, at both the transcriptional and epigenetic levels. These modifications influence various processes, such as metabolism, immune system function, and responses to pathogens [35–40].

Previous research has shown that certain bacterial proteins can alter host signaling molecules at the posttranscriptional level, enabling bacterial invasion and promoting their replication within the host [41, 42]. For example, studies have demonstrated that proteins such as RomA from *Legionella pneumophila*, NUE from *Chlamydia trachomatis*, and BaSET from *Bacillus anthracis* can methylate histones, thereby modulating host immune responses to these pathogens [43–48]. Furthermore, the *Listeria monocytogenes* protein InlB has been shown to induce the deacetylation of H3K18 in a SIRT2-dependent manner [49]. In our previous study, we demonstrated for the first time that α-hemolysin from *S. aureus* alters the methylation patterns in the genomes of human Th17 lymphocytes [10]. This finding prompted us to conduct a similar analysis on Th1 cells, which are also involved in the immune response to this pathogen [50]. RNA-seq analysis revealed that the expression of numerous genes, including those involved in the immune response, was altered in Th1 lymphocytes following treatment with α-hemolysin (Figs. 2 and 3). Since α-hemolysin has also been shown to affect the protein levels of specific targets, such as DNMT3B [10], we further examined the expression of DNA methyltransferases via western blotting. This analysis revealed that sublytic concentrations of α-hemolysin led to changes in the protein levels of HELLS, DNMT3A, and DNMT3L (Fig. 4). Unlike Th17 lymphocytes, Th1 cells presented relatively subtle changes in CG methylation, whereas more pronounced effects were observed for non-CpG island-associated methylation (CHG and CHH) (Fig. 5, 6, 7, 9 and 10); we speculate that this may be linked to the increase in HELLS and DNMT3A levels and decrease in the levels of DNMT3L, proteins that interact with each other and are involved in different types of DNA methylation [51–56]. However, we should also highlight the differences in the expression of proteins involved in DNA methylation between Th1 and Th17 cells. Specifically, DNMT3B expression was not detected in Th1 lymphocytes, whereas in Th17 cells, this protein was clearly downregulated by hemolysin and may be responsible for the observed changes in DNA methylation [10]. These findings indicate that different mechanisms are likely responsible for the changes in DNA methylation in these two T-cell subpopulations; this is supported by the observation that both subpopulations exhibit different sensitivities to α-hemolysin, as shown here and previously reported by other authors [27]. These findings suggest that variations in the repertoire of the cellular receptors involved in the response to this toxin or differences in the cellular membrane composition may play a role [57, 58]. However, further investigation is needed, as the differences in the immune cell response cannot be solely attributed to variations in the expression of the α-hemolysin receptor ADAM10 [27].

CHG and CHH methylation cannot be maintained and therefore depend on de novo methylation and enzymes such as DNMT3s [59–61]. Although extensively studied in plants, methylation outside CpG islands in animals has received less attention, despite potentially accounting for over 50% of methylated cytosines [62] and being frequently associated with transcribed genes, particularly within intragenic regions [34, 63, 64]. The general function of these DNA modifications, however, remains unclear [65, 66], although many studies suggest that methylation at non-CpG sites may be associated with reduced gene expression [67, 68], as these sites are bound by methyl-CpG binding protein 2 (MeCP2) [68, 69]. However, the precise impact of this type of methylation, which was also analyzed in this study, on gene expression remains unclear, as not all methylation sites contribute equally to the regulation of gene expression [70–72]. Notably, we found that *staphylococcal* α-hemolysin increased HELLS and DNMT3A protein expression, decreased DNMT3L expression (Fig. 4), and influenced the methylation of numerous genomic regions (Figs. 5, 6, 7, 8, 9 and 10). This effect may be linked to the cellular response to stress stimuli triggered by a bacterial protein. This connection is partially supported by the GO analysis results (Table 2), which revealed that 'anchored component of the plasma membrane'and 'calcium ion binding' were enriched in the DMR genes. It is well established that α-hemolysin targets cellular membranes and disrupts the calcium ion balance within cells [9]; moreover, it could play a role in maintaining genomic stability or leaving an epigenetic mark on cells to increase their plasticity and/or modify immunological responses following contact with the pathogen, as suggested by the other enriched GO terms (Table 2). This was previously suggested by Mba Medie et al., who reported that bacteremia caused by *S. aureus* leads to a decrease in DNMT3A levels and alterations in DNA methylation patterns in human macrophages. This was associated with increased susceptibility of patients to the bacterium, suggesting that this methyltransferase plays a role in protective immune responses [73].

In summary, our study demonstrated that the interaction between bacterial proteins and the host can induce transcriptional and epigenetic changes in the host, potentially marking host cells as having already been in contact with this bacterium; this may result in notable alterations in cellular phenotypes and immune responses, potentially affecting the ability of the immune system to defend against pathogens in the future.

The RNA-seq and WGBS results are available under the BioProject accession number at the NCBI Short-Read Archive (SRA): PRJNA1254664 and PRJNA1146149 respectively.

## Supplementary information

Below is the link to the electronic supplementary material.

ESM 1 (DOCX 1.52 MB)

ESM 2 (XLS 1.33 MB)

ESM 3 (XLS 4.04 MB)

## Data Availability

The RNA-seq and WGBS results are available under the BioProject accession number at the NCBI Short-Read Archive (SRA): PRJNA1254664 and PRJNA1146149 respectively.
