# Supplementary material for: Staphylococcus aureus α-hemolysin induces DNA methylation changes in human Th1 cells
Source: Immunol Res. 2025 Jun 11;73(1):95. doi: 10.1007/s12026-025-09647-0 (PMC12159126; doi:10.1007/s12026-025-09647-0)
Supplement: Supplementary file 1 — (DOCX 1.52 MB) [file 12026_2025_9647_MOESM1_ESM.docx]

Supplementary Materials

*Staphylococcus aureus* α-hemolysin induces DNA methylation changes in human Th1 cells.

Iwona Karwaciak^1^^, Joanna Pastwińska^1^^, Anna Sałkowska^1^, Kaja Karaś^1^, Marta Sobalska-Kwapis^2^, Jarosław Dastych^3^ and Marcin Ratajewski^1*^

^^^ Contributed equally

^1^ Laboratory of Epigenetics, Institute of Medical Biology, Polish Academy of Sciences, Lodowa 106, 93-232, Lodz, Poland.

^2^ Centre for Digital Biology and Biomedical Science - Biobank Lodz®, Faculty of Biology and Environmental Protection, University of Lodz, 91-402, Lodz, Poland.

^3^ Laboratory of Cellular Immunology, Institute of Medical Biology, Polish Academy of Sciences, Lodowa 106, 93-232, Lodz, Poland.


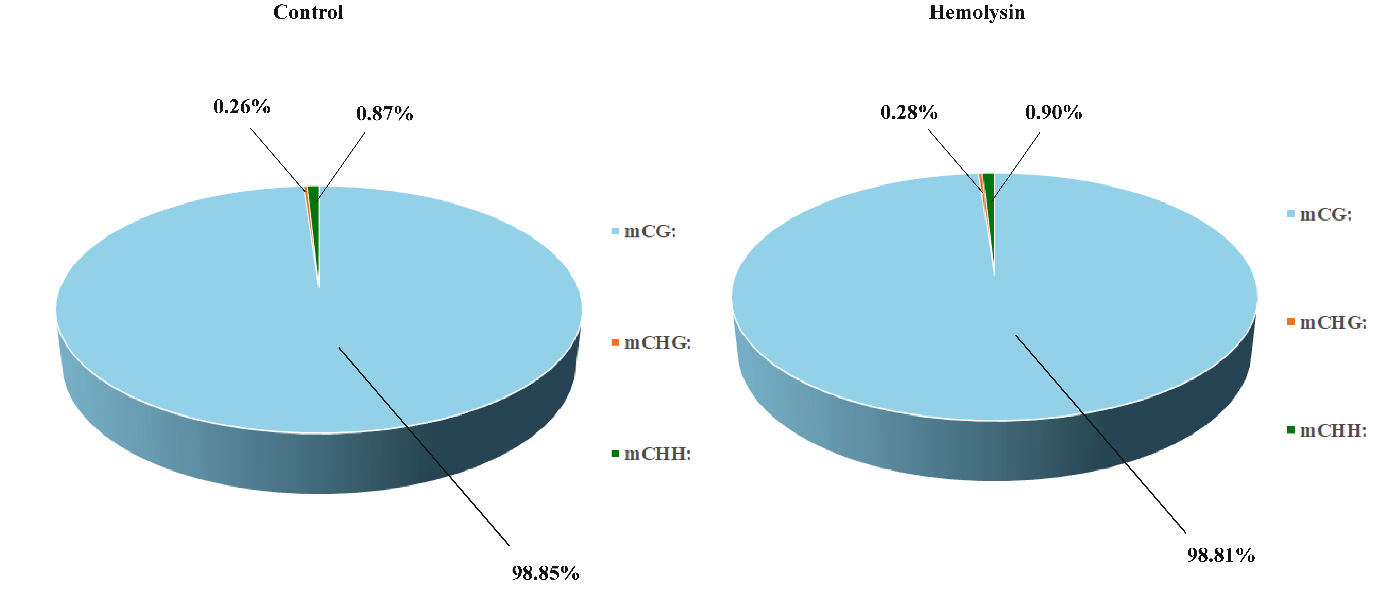


Figure S1. The average ratio of DNA methylation types in the genomes of control and α-hemolysin-treated Th1 lymphocytes was determined via WGBS of cells originating from five different donors. The pie chart uses colors to represent different methylation types, while the numbers indicate the percentage corresponding to each type.


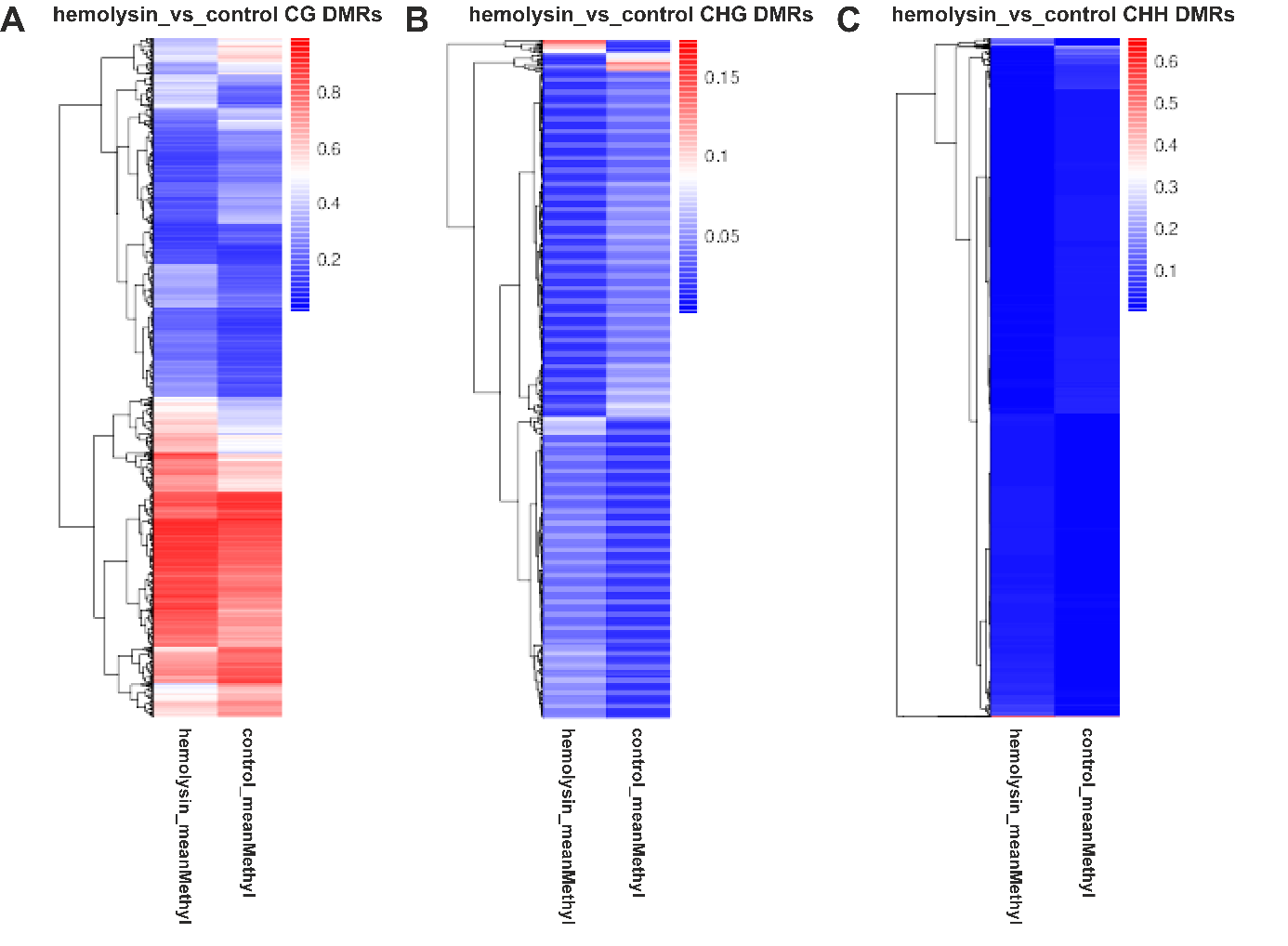


Figure S2. Cluster heatmaps for DMR methylation levels in the CG (A), CHG (B) and CHH (C) contexts.

Table S1. Primer pairs used to detect the mRNAs of cognate genes.

| Gene | Primer forward | Primer reverse |
| --- | --- | --- |
| *IFNG* | 5’-CAGGTCATTCAGATGTAGCGG-3’ | 5’-CATGTATTGCTTTGCGTTGG-3’ |
| *IL2* | 5'-TTTTACATGCCCAAGAAGGC-3' | 5'-ATGGTTGCTGTCTCATCAGC-3' |
| *IL6* | 5'-CCTGAACCTTCCAAAGATGG-3' | 5'-GGTCAGGGGTGGTTATTGC-3' |
| *IL13* | 5'-TGAGGAGCTGGTCAACATCA-3' | 5'-CAGGTTGATGCTCCATACC-3' |
| *GZMB* | 5'- TCAAAGAACAGGAGCCGACC-3' | 5'- TGTGAGTGTTTTCCCAGGGG -3' |
| *HELLS* | 5'-CTGGAGGAGTGATGCGATGG-3' | 5'-GGGCCACAGACAAGAAAAGG-3' |
| *HPRT1* | 5'- TGACACTGGCAAAACAATGCA-3' | 5'- GGTCCTTTTCACCAGCAAGCT-3' |
| *HMBS* | 5'- GGCAATGCGGCTGCAA-3' | 5'- GGGTACCCACGCGAATCAC-3' |
| *RPL13A* | 5'-CCTGGAGGAGAAGAGGAAAGAGA-3' | 5'- TTGAGGACCTCTGTGTATTTGTCAA -3' |

Table S2. GOseq analysis results for the significant DEGs (biological process, cellular component, and molecular function terms) after α-hemolysin treatment in Th1 cells.

Biological Process

| **Enrichment FDR** | **nGenes** | **Pathway Genes** | **Fold Enrichment** | **Pathways** |
| --- | --- | --- | --- | --- |
| 7.1E-11 | 21 | 39 | 6.7 | Maturation of 5.8S rRNA |
| 3.0E-27 | 83 | 248 | 4.2 | RRNA processing |
| 8.2E-33 | 105 | 327 | 4 | Ribosome biogenesis |
| 7.7E-26 | 88 | 288 | 3.8 | RRNA metabolic proc. |
| 2.1E-29 | 129 | 509 | 3.2 | Ribonucleoprotein complex biogenesis |
| 6.9E-25 | 117 | 483 | 3 | NcRNA processing |
| 1.7E-23 | 137 | 645 | 2.6 | NcRNA metabolic proc. |
| 3.6E-13 | 90 | 450 | 2.5 | Response to virus |
| 2.1E-10 | 83 | 449 | 2.3 | Reg. of translation |
| 5.8E-11 | 95 | 533 | 2.2 | Reg. of cellular macromolecule biosynthetic proc. |
| 2.1E-10 | 91 | 514 | 2.2 | Reg. of cellular amide metabolic proc. |
| 9.2E-22 | 188 | 1082 | 2.2 | RNA processing |
| 4.4E-11 | 125 | 784 | 2 | Translation |
| 2.0E-13 | 172 | 1138 | 1.9 | DNA metabolic proc. |
| 7.1E-11 | 148 | 999 | 1.8 | Peptide metabolic proc. |
| 1.6E-10 | 179 | 1301 | 1.7 | Cellular amide metabolic proc. |
| 5.3E-11 | 241 | 1888 | 1.6 | Organonitrogen compound biosynthetic proc. |
| 2.7E-12 | 314 | 2575 | 1.5 | Reg. of cellular protein metabolic proc. |
| 4.1E-13 | 339 | 2795 | 1.5 | Reg. of protein metabolic proc. |
| 5.8E-13 | 417 | 3630 | 1.4 | Cellular component biogenesis |

Cellular Component

| **Enrichment FDR** | **nGenes** | **Pathway Genes** | **Fold Enrichment** | **Pathways** |
| --- | --- | --- | --- | --- |
| 1.5E-08 | 15 | 26 | 7.2 | Preribosome large subunit precursor |
| 5.6E-05 | 9 | 16 | 7 | Nuclear exosome (RNase complex) |
| 1.6E-07 | 15 | 30 | 6.2 | 90S preribosome |
| 7.2E-05 | 11 | 25 | 5.5 | Exoribonuclease complex |
| 6.4E-16 | 37 | 88 | 5.2 | Preribosome |
| 1.3E-16 | 141 | 807 | 2.2 | Ribonucleoprotein complex |
| 3.1E-22 | 193 | 1126 | 2.1 | Nucleolus |
| 1.4E-04 | 62 | 423 | 1.8 | Chromosomal region |
| 2.0E-05 | 80 | 559 | 1.8 | Mitochondrial inner membrane |
| 5.6E-05 | 74 | 520 | 1.8 | Mitochondrial matrix |
| 1.7E-05 | 88 | 630 | 1.7 | Organelle inner membrane |
| 4.4E-09 | 191 | 1489 | 1.6 | Nuclear protein-containing complex |
| 1.8E-08 | 177 | 1380 | 1.6 | Organelle envelope |
| 1.8E-08 | 177 | 1380 | 1.6 | Envelope |
| 3.0E-11 | 234 | 1830 | 1.6 | Mitochondrion |
| 4.1E-05 | 112 | 879 | 1.6 | Mitochondrial envelope |
| 1.1E-26 | 599 | 4973 | 1.5 | Nuclear lumen |
| 6.7E-23 | 547 | 4581 | 1.5 | Nucleoplasm |
| 1.9E-05 | 169 | 1441 | 1.5 | Microtubule cytoskeleton |
| 2.8E-06 | 227 | 2003 | 1.4 | Chromosome |

Molecular Function

| **Enrichment FDR** | **nGenes** | **Pathway Genes** | **Fold Enrichment** | **Pathways** |
| --- | --- | --- | --- | --- |
| 4.3E-11 | 84 | 455 | 2.3 | Catalytic activity acting on RNA |
| 1.6E-43 | 330 | 1852 | 2.2 | RNA binding |
| 1.2E-12 | 116 | 687 | 2.1 | Catalytic activity acting on a nucleic acid |
| 9.1E-13 | 121 | 724 | 2.1 | Nucleoside-triphosphatase activity |
| 1.1E-12 | 128 | 787 | 2 | Pyrophosphatase activity |
| 1.2E-12 | 128 | 790 | 2 | Hydrolase activity acting on acid anhydrides |
| 7.5E-17 | 312 | 2381 | 1.6 | Nucleotide binding |
| 7.5E-17 | 312 | 2382 | 1.6 | Nucleoside phosphate binding |
| 3.8E-14 | 266 | 2034 | 1.6 | Purine ribonucleoside triphosphate binding |
| 1.0E-14 | 277 | 2120 | 1.6 | Purine nucleotide binding |
| 3.9E-14 | 273 | 2106 | 1.6 | Purine ribonucleotide binding |
| 5.4E-14 | 274 | 2123 | 1.6 | Ribonucleotide binding |
| 1.9E-10 | 213 | 1662 | 1.6 | ATP binding |
| 8.4E-16 | 320 | 2505 | 1.6 | Carbohydrate derivative binding |
| 1.1E-10 | 222 | 1741 | 1.6 | Adenyl nucleotide binding |
| 2.7E-10 | 219 | 1729 | 1.6 | Adenyl ribonucleotide binding |
| 5.0E-15 | 340 | 2743 | 1.5 | Small molecule binding |
| 1.2E-11 | 313 | 2630 | 1.5 | Anion binding |
| 4.1E-20 | 520 | 4400 | 1.5 | Nucleic acid binding |

Table S3. KEGG pathway analysis revealed pathways significantly associated with differentially expressed genes (DEGs) in differentiating Th1 lymphocytes following α-hemolysin treatment.

| **Enrichment FDR** | **nGenes** | **Pathway Genes** | **Fold Enrichment** | **Pathways** |
| --- | --- | --- | --- | --- |
| 3.7E-03 | 11 | 36 | 3.8 | DNA replication |
| 6.3E-06 | 23 | 77 | 3.7 | Ribosome biogenesis in eukaryotes |
| 7.7E-03 | 10 | 34 | 3.7 | RNA polymerase |
| 3.4E-02 | 8 | 30 | 3.3 | Antifolate resistance |
| 5.5E-03 | 14 | 58 | 3 | Pyrimidine metabolism |
| 2.3E-02 | 11 | 47 | 2.9 | Other types of O-glycan biosynthesis |
| 1.8E-04 | 25 | 108 | 2.9 | Nucleocytoplasmic transport |
| 9.6E-03 | 14 | 62 | 2.8 | Viral life cycle-HIV-1 |
| 2.6E-02 | 13 | 63 | 2.6 | Cytosolic DNA-sensing pathway |
| 4.9E-04 | 27 | 131 | 2.6 | Spliceosome |
| 2.3E-02 | 16 | 85 | 2.3 | Nucleotide metabolism |
| 4.6E-02 | 16 | 93 | 2.1 | IL-17 signaling pathway |
| 3.9E-03 | 29 | 171 | 2.1 | Influenza A |
| 1.4E-02 | 23 | 136 | 2.1 | Apoptosis |
| 1.9E-02 | 25 | 157 | 2 | Hepatitis C |
| 1.4E-03 | 45 | 294 | 1.9 | Cytokine-cytokine receptor interaction |
| 1.4E-03 | 53 | 364 | 1.8 | Amyotrophic lateral sclerosis |
| 2.9E-02 | 29 | 202 | 1.8 | Epstein-Barr virus infection |
| 1.7E-03 | 64 | 476 | 1.7 | Pathways of neurodegeneration-multiple diseases |
| 2.8E-02 | 48 | 384 | 1.6 | Alzheimer disease |

Table S4. Differentially expressed genes (DEGs) whose promoters were hypermethylated or hypomethylated following α-hemolysin treatment in Th1 lymphocytes.

| DEG | Name | Promoter/context |
| --- | --- | --- |
| ENSG00000009335 | UBE3C | Hypermethylion/CG |
| ENSG00000012779 | ALOX5 | Hypermethylation/CG |
| ENSG00000052749 | RRP12 | Hypermethylation/CG |
| ENSG00000054793 | ATP9A | Hypermethylation/CG |
| ENSG00000056487 | PHF21B | Hypermethylation/CG |
| ENSG00000066629 | EML1 | Hypermethylation/CG |
| ENSG00000067057 | PFKP | Hypermethylation/CG |
| ENSG00000068976 | PYGM | Hypermethylation/CHH |
| ENSG00000071242 | RPS6KA2 | Hypermethylation/CG |
| ENSG00000075043 | KCNQ2 | Hypermethylation/CG |
| ENSG00000087258 | GNAO1 | Hypermethylation/CG |
| ENSG00000100034 | PPM1F | Hypermethylation/CHH |
| ENSG00000100320 | RBFOX2 | Hypermethylation/CG |
| ENSG00000100591 | AHSA1 | Hypermethylation/CHH |
| ENSG00000101203 | COL20A1 | Hypermethylation/CHG |
| ENSG00000101246 | ARFRP1 | Hypermethylation/CG |
| ENSG00000104524 | PYCR3 | Hypermethylation/CHH |
| ENSG00000104824 | HNRNPL | Hypermethylation/CG |
| ENSG00000104885 | DOT1L | Hypermethylation/CHH |
| ENSG00000104907 | TRMT1 | Hypermethylation/CG |
| ENSG00000105173 | CCNE1 | Hypermethylation/CG |
| ENSG00000105576 | TNPO2 | Hypermethylation/CG |
| ENSG00000106070 | GRB10 | Hypermethylation/CG |
| ENSG00000106263 | EIF3B | Hypermethylation/CHH |
| ENSG00000108312 | UBTF | Hypermethylation/CG |
| ENSG00000111328 | CDK2AP1 | Hypermethylation/CG |
| ENSG00000111331 | OAS3 | Hypermethylation/CHH |
| ENSG00000115129 | TP53I3 | Hypermethylation/CHH |
| ENSG00000116954 | RRAGC | Hypermethylation/CG |
| ENSG00000117707 | PROX1 | Hypermethylation/CG |
| ENSG00000118965 | WDR35 | Hypermethylation/CHH |
| ENSG00000119335 | SET | Hypermethylation/CG |
| ENSG00000119630 | PGF | Hypermethylation/CHG |
| ENSG00000120278 | PLEKHG1 | Hypermethylation/CG |
| ENSG00000120705 | ETF1 | Hypermethylation/CHH |
| ENSG00000121060 | TRIM25 | Hypermethylation/CHH |
| ENSG00000124299 | PEPD | Hypermethylation/CHH |
| ENSG00000127418 | FGFRL1 | Hypermethylation/CG |
| ENSG00000128268 | MGAT3 | Hypermethylation/CG |
| ENSG00000129173 | E2F8 | Hypermethylation/CG |
| ENSG00000134247 | PTGFRN | Hypermethylation/CG |
| ENSG00000134802 | SLC43A3 | Hypermethylation/CHG |
| ENSG00000135314 | KHDC1 | Hypermethylation/CHH |
| ENSG00000135862 | LAMC1 | Hypermethylation/CG |
| ENSG00000141232 | TOB1 | Hypermethylation/CG |
| ENSG00000141376 | BCAS3 | Hypermethylation/CG |
| ENSG00000141522 | ARHGDIA | Hypermethylation/CG |
| ENSG00000149136 | SSRP1 | Hypermethylation/CHG |
| ENSG00000152061 | RABGAP1L | Hypermethylation/CHH |
| ENSG00000152413 | HOMER1 | Hypermethylation/CG |
| ENSG00000153207 | AHCTF1 | Hypermethylation/CG |
| ENSG00000157087 | ATP2B2 | Hypermethylation/CG |
| ENSG00000159399 | HK2 | Hypermethylation/CHH |
| ENSG00000163013 | FBXO41 | Hypermethylation/CHH |
| ENSG00000163412 | EIF4E3 | Hypermethylation/CG |
| ENSG00000164362 | TERT | Hypermethylation/CG |
| ENSG00000166166 | TRMT61A | Hypermethylation/CHH |
| ENSG00000166833 | NAV2 | Hypermethylation/CHH |
| ENSG00000170835 | CEL | Hypermethylation/CG |
| ENSG00000172301 | COPRS | Hypermethylation/CG |
| ENSG00000172348 | RCAN2 | Hypermethylation/CHH |
| ENSG00000172889 | EGFL7 | Hypermethylation/CG |
| ENSG00000173175 | ADCY5 | Hypermethylation/CG |
| ENSG00000173404 | INSM1 | Hypermethylation/CG |
| ENSG00000175773 | ZBTB44-DT | Hypermethylation/CG |
| ENSG00000176170 | SPHK1 | Hypermethylation/CHG |
| ENSG00000178409 | BEND3 | Hypermethylation/CG |
| ENSG00000178752 | ERFE | Hypermethylation/CG |
| ENSG00000178921 | PFAS | Hypermethylation/CG |
| ENSG00000183474 | GTF2H2C | Hypermethylation/CHH |
| ENSG00000185158 | LRRC37B | Hypermethylation/CG |
| ENSG00000186665 | C17orf58 | Hypermethylation/CG |
| ENSG00000187010 | RHD | Hypermethylation/CHH |
| ENSG00000188807 | TMEM201 | Hypermethylation/CG |
| ENSG00000189184 | PCDH18 | Hypermethylation/CHH |
| ENSG00000196876 | SCN8A | Hypermethylation/CG |
| ENSG00000197905 | TEAD4 | Hypermethylation/CG |
| ENSG00000198176 | TFDP1 | Hypermethylation/CG |
| ENSG00000198498 | TMA16 | Hypermethylation/CG |
| ENSG00000198752 | CDC42BPB | Hypermethylation/CG |
| ENSG00000226742 | HSBP1L1 | Hypermethylation/CG |
| ENSG00000228140 | EFHD2-AS1 | Hypermethylation/CG |
| ENSG00000234884 | GRK3-AS1 | Hypermethylation/CG |
| ENSG00000235159 | LINC02939 | Hypermethylation/CG |
| ENSG00000239697 | TNFSF12 | Hypermethylation/CG |
| ENSG00000265660 | MIR4664 | Hypermethylation/CG |
| ENSG00000276203 | ANKRD20A3P | Hypermethylation/CG |
| ENSG00000278619 | MRM1 | Hypermethylation/CHH |
| ENSG00000007541 | PIGQ | Hypomethylation/CHH |
| ENSG00000009830 | POMT2 | Hypomethylation/CHH |
| ENSG00000011304 | PTBP1 | Hypomethylation/CG |
| ENSG00000059378 | PARP12 | Hypomethylation/CHH |
| ENSG00000063660 | GPC1 | Hypomethylation/CG |
| ENSG00000064545 | TMEM161A | Hypomethylation/CHH |
| ENSG00000066697 | MSANTD3 | Hypomethylation/CG |
| ENSG00000070423 | RNF126 | Hypomethylation/CG |
| ENSG00000070614 | NDST1 | Hypomethylation/CHH |
| ENSG00000071242 | RPS6KA2 | Hypomethylation/CG |
| ENSG00000088256 | GNA11 | Hypomethylation/CHG |
| ENSG00000101255 | TRIB3 | Hypomethylation/CG |
| ENSG00000101361 | NOP56 | Hypomethylation/CG |
| ENSG00000104824 | HNRNPL | Hypomethylation/CG |
| ENSG00000106070 | GRB10 | Hypomethylation/CG |
| ENSG00000108306 | FBXL20 | Hypomethylation/CG |
| ENSG00000108312 | UBTF | Hypomethylation/CHH |
| ENSG00000111328 | CDK2AP1 | Hypomethylation/CG |
| ENSG00000117676 | RPS6KA1 | Hypomethylation/CHH |
| ENSG00000118515 | SGK1 | Hypomethylation/CHH |
| ENSG00000119943 | PYROXD2 | Hypomethylation/CHH |
| ENSG00000123131 | PRDX4 | Hypomethylation/CG |
| ENSG00000124191 | TOX2 | Hypomethylation/CG |
| ENSG00000124535 | WRNIP1 | Hypomethylation/CG |
| ENSG00000129990 | SYT5 | Hypomethylation/CHH |
| ENSG00000130382 | MLLT1 | Hypomethylation/CG |
| ENSG00000130487 | KLHDC7B | Hypomethylation/CG |
| ENSG00000130939 | UBE4B | Hypomethylation/CHH |
| ENSG00000134198 | TSPAN2 | Hypomethylation/CG |
| ENSG00000135316 | SYNCRIP | Hypomethylation/CG |
| ENSG00000139372 | TDG | Hypomethylation/CHG |
| ENSG00000140750 | ARHGAP17 | Hypomethylation/CHG |
| ENSG00000144535 | DIS3L2 | Hypomethylation/CHH |
| ENSG00000147576 | ADHFE1 | Hypomethylation/CHH |
| ENSG00000151413 | NUBPL | Hypomethylation/CHH |
| ENSG00000158716 | DUSP23 | Hypomethylation/CHH |
| ENSG00000159216 | RUNX1 | Hypomethylation/CG |
| ENSG00000161980 | POLR3K | Hypomethylation/CHG |
| ENSG00000161981 | SNRNP25 | Hypomethylation/CHG |
| ENSG00000162337 | LRP5 | Hypomethylation/CG |
| ENSG00000162614 | NEXN | Hypomethylation/CHH |
| ENSG00000163132 | MSX1 | Hypomethylation/CHH |
| ENSG00000168453 | HR | Hypomethylation/CG |
| ENSG00000170017 | ALCAM | Hypomethylation/CHH |
| ENSG00000170153 | RNF150 | Hypomethylation/CG |
| ENSG00000172336 | POP7 | Hypomethylation/CHH |
| ENSG00000173457 | PPP1R14B | Hypomethylation/CG |
| ENSG00000174672 | BRSK2 | Hypomethylation/CG |
| ENSG00000177189 | RPS6KA3 | Hypomethylation/CHH |
| ENSG00000178409 | BEND3 | Hypomethylation/CG |
| ENSG00000180539 | LINC02908 | Hypomethylation/CHH |
| ENSG00000182612 | TSPAN10 | Hypomethylation/CG |
| ENSG00000187682 | ERAS | Hypomethylation/CG |
| ENSG00000188976 | NOC2L | Hypomethylation/CG |
| ENSG00000196715 | VKORC1L1 | Hypomethylation/CG |
| ENSG00000215158 | GUSBP18 | Hypomethylation/CG |
| ENSG00000215859 | PDZK1P1 | Hypomethylation/CG |
| ENSG00000226067 | LINC00623 | Hypomethylation/CG |
| ENSG00000235531 | MSC-AS1 | Hypomethylation/CHH |
| ENSG00000237276 | ANO7L1 | Hypomethylation/CHH |
| ENSG00000250508 | LINC02701 | Hypomethylation/CG |
| ENSG00000271503 | CCL5 | Hypomethylation/CHH |
| ENSG00000275835 | TUBGCP5 | Hypomethylation/CG |
| ENSG00000275896 | PRSS2 | Hypomethylation/CHH |
| ENSG00000278259 | MYO19 | Hypomethylation/CHH |


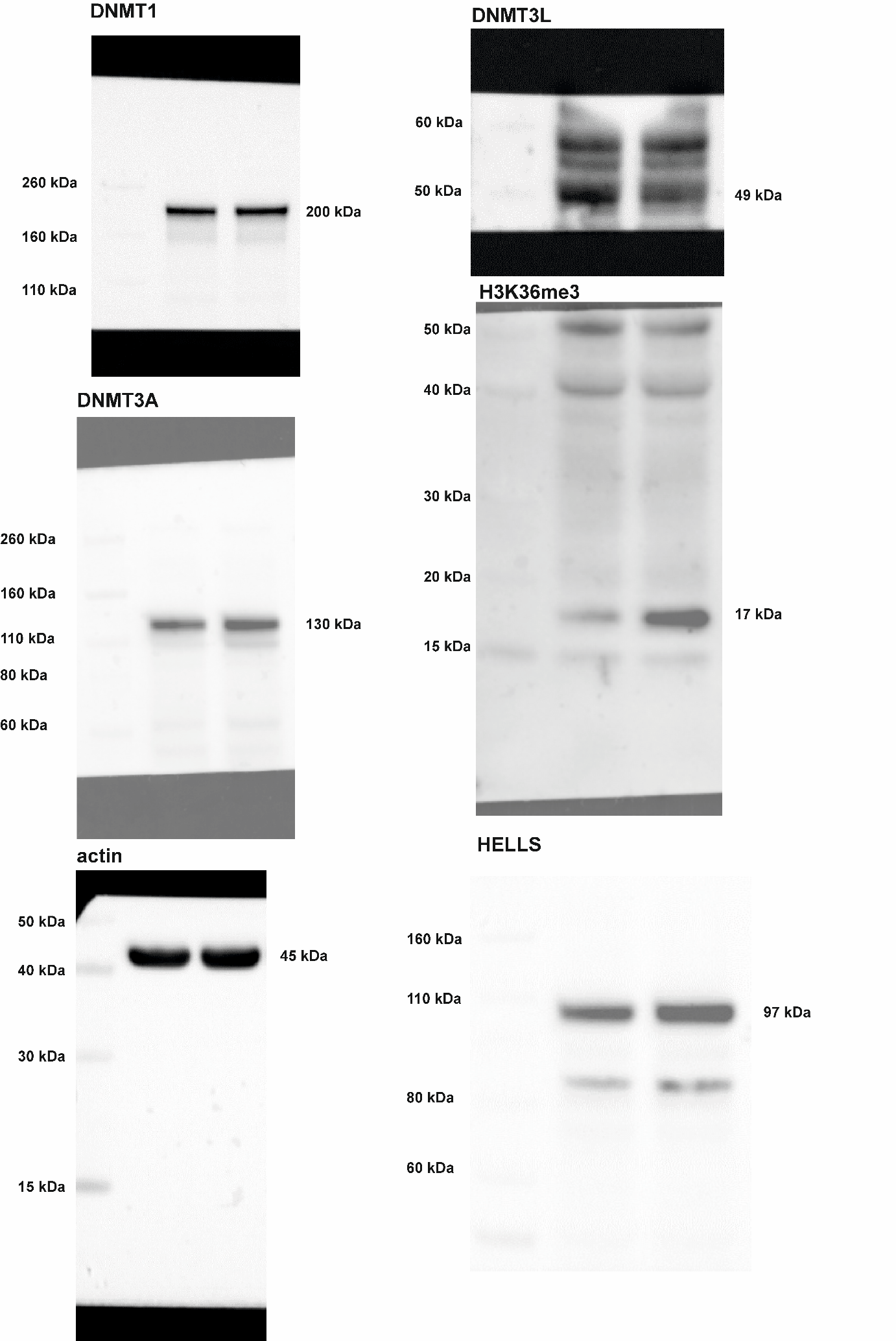


Figure S3. Original western blot scans.
